# Editorial: Mapping symptom networks among co-occurrence of psychological and somatic symptoms

**DOI:** 10.3389/fpubh.2023.1210151

**Published:** 2023-05-23

**Authors:** Zheng Zhu, Hui Hu, Bei Wu, Yan Hu

**Affiliations:** ^1^School of Nursing, Fudan University, Shanghai, China; ^2^Fudan University Centre for Evidence-based Nursing: A Joanna Briggs Institute Centre of Excellence, Fudan University, Shanghai, China; ^3^NYU Rory Meyers College of Nursing, New York University, New York, NY, United States; ^4^Channing Division of Network Medicine, Department of Medicine, Brigham and Women's Hospital and Harvard Medical School, Boston, MA, United States

**Keywords:** complex network, symptom network, symptom, phenotype, psychopathology

Psychological and somatic symptoms often co-occur in individuals with mental health and physical health conditions. Previous studies have shown that more than 30–50% of people with cancer, chronic non-communicable diseases, infectious diseases, or mental disorders had an average of seven or more psychological and somatic symptoms at the same time (1). These symptoms can interact in complex ways, leading to significant impairment in quality of life and functioning. Understanding the relationships between co-occurring psychological and somatic symptoms is important for developing effective interventions to address these conditions (2).

Despite extensive research, there is still a limited understanding of the complex interactions between psychological and somatic symptoms, as well as how these relationships may change or evolve over time. This lack of knowledge presents significant challenges in treating individuals who experience multiple symptoms simultaneously, as the connections among the various symptoms are often uncertain and intricate. The task of diagnosing and treating such individuals is further complicated by the fact that symptoms are typically not the result of a single disease or the dysfunction of just one effector gene product. Rather, they are often the outcome of a multitude of interconnected pathobiological processes working together in a sophisticated and dynamic network. This complexity highlights the need for a more comprehensive approach to understanding the underlying mechanisms and interactions of symptoms.

In the context of data-intensive biomedicine's new paradigm that emphasizes the interconnectedness of symptoms, novel inference techniques are emerging. Based on complex network theory, mapping symptom networks has gained popularity in recent years. By mapping out networks of symptoms based on their co-occurrence in psychological and somatic contexts, we gain insight into the internal structure of disease phenotypes (3). This visualization of symptom interactions can facilitate researchers in developing more precise and personalized interventions.

This Research Topic aims to build and analyze symptom networks based on clinical real-world data (e.g., symptoms, diseases, and phenotypic data). The issue currently includes five papers that explore the mechanisms of empathy and symptoms of depression among university students during the COVID-19 pandemic, identify the central and bridging psychological symptoms of people living with HIV, characterize the somatic symptom network structure in patients with depressive disorders, explore core mental health symptoms in people living with HIV, and examine the complex relationship of endocrine therapy-related symptoms.

Exploring core symptoms, bridge symptoms, and internal structure of symptoms are major functions for mapping symptom networks. The indicators for networks and nodes may provide empirical evidence for the target of symptom management interventions. Wen et al. investigated the psychological symptom network of people living with HIV and identified the central and bridge symptoms. The results showed that sadness was the central symptom in people living with HIV, and three bridge symptoms existed among different clusters of psychological symptoms. Based on network theory, both central and bridge symptoms can represent promising intervention targets from different perspectives. In addition, Han et al. evaluated the prevalence of mental health symptoms and explored their relationships among people living with HIV through network analysis. The study recruited 518 participants through convenience sampling in Beijing, China, and assessed 40 mental health symptoms across six dimensions. The study found that negative affect was the most central symptom cluster, while sadness, feeling discouraged about the future, and feelings of worthlessness were the most central symptoms. The findings provide insight into the core mental health symptoms and clusters experienced by people living with HIV, which can help guide tailored interventions to improve their mental health and wellbeing.

Symptom networks have increasingly been shown to be a promising approach for exploring the mechanism between domains under one construct. Li J. et al. used network analysis to examine the relationship between dimensions of empathy and symptoms of depression among university students during the COVID-19 pandemic. The study found that personal distress was positively linked to symptoms of depression, while empathic concern was negatively associated with suicidal thoughts and psychomotor agitation or retardation. The study also identified influential nodes that bridge empathy and depression and found no significant sex differences in the structure of the empathy-depression network. The study's findings contribute to our understanding of the complex relationship between empathy and depression and may inform interventions and preventions for depressive symptoms during the pandemic.

Focusing on the co-occurrence of somatic and psychological symptoms may help researchers better understand the mechanism between mind and body. It can provide insight into how psychological factors such as stress, anxiety, and depression can manifest as physical symptoms and how physical health conditions can impact psychological wellbeing. Li Y. et al. aimed to analyze the somatic symptom network of patients with depressive disorder using the Patient Health Questionnaire. A total of 177 patients diagnosed with depressive disorder were included in the study. The findings indicated that feeling one's heart pound or race, shortness of breath, and back pain were the most central somatic symptoms, highly connected with the remaining somatic symptoms, and could trigger or maintain them. Clinicians should focus more on palpitations and chest tightness and increase efforts toward early identification and targeted model management to ensure improvement in somatic symptoms and treatment outcomes. Jing et al. aimed to explore the symptom network of breast cancer patients undergoing endocrine therapy and identify the most central symptoms. Data from 613 breast cancer patients in Shanghai, China, were analyzed using network analysis techniques. The results showed that irritability and mood swings were the most prevalent and central symptoms. Developing interventions targeting emotional symptoms may be crucial in reducing the overall symptom burden among breast cancer patients undergoing endocrine therapy.

In conclusion, this Research Topic collects cutting-edge contributions in the area of network science and provides new evidence for understanding the interactions among co-occurring psychological and somatic symptoms. The following aspects are provided as references for future studies in this area:

- Dynamic network analysis using longitudinal data: Longitudinal data can provide valuable information on how symptom networks change over time. Future studies could use dynamic network analysis to investigate changes in the network structure of co-occurring psychological and somatic symptoms over time. This approach could provide insights into the temporal dynamics of symptom networks and help identify critical time points for intervention.
- Investigating the heterogeneity of symptom networks in different subgroups: Co-occurring psychological and somatic symptoms may differ across different populations, such as different age groups, genders, or cultural backgrounds. Future studies could explore the heterogeneity of symptom networks in different subgroups to better understand how symptom networks vary across different populations. This could inform more tailored and targeted interventions.
- Combining symptomics with real-world big data in the clinical setting: Advances in data science and technology have opened up new opportunities for combining symptomics with real-world big data in the clinical setting. Future studies could explore how integrating symptomics with real-world big data, such as electronic health records, wearable sensors, and social media data, could enhance our understanding of co-occurring psychological and somatic symptoms. This could lead to more personalized and precision medicine approaches to treatment.

## Author contributions

ZZ was a guest associate editor of the Research Topic and wrote the paper text. HH, BW, and YH acted as editor in the Research Topic. All authors contributed to the article and approved the submitted version.
